# Chromatographic Determination and Antimicrobial Evaluation of Walnut (*Juglans regia* L.) Septa from Different Habitats

**DOI:** 10.3390/plants15081263

**Published:** 2026-04-20

**Authors:** Jurgita Luksiene, Nerija Zevzikovaite, Jurga Andreja Kazlauskaite, Mindaugas Marksa, Agne Giedraitiene, Lina Merkeviciene, Asta Kubiliene, Andrejus Zevzikovas

**Affiliations:** 1 Department of Analytical and Toxicological Chemistry, Lithuanian University of Health Sciences, Sukileliu pr. 13, LT-50161 Kaunas, Lithuania; nerija.zevzikovaite@stud.lsmu.lt (N.Z.); mindaugas.marksa@lsmu.lt (M.M.); asta.kubiliene@lsmu.lt (A.K.); andrejus.zevzikovas@lsmu.lt (A.Z.); 2 Department of Drug Technology and Social Pharmacy, Lithuanian University of Health Sciences, Sukileliu pr. 13, LT-50161 Kaunas, Lithuania; jurga.andreja.kazlauskaite@lsmu.lt; 3 Institute of Pharmaceutical Technologies, Lithuanian University of Health Sciences, LT-50161 Kaunas, Lithuania; 4 Institute of Microbiology and Virology, Faculty of Veterinary Medicine, Lithuanian University of Health Sciences, Mickeviciaus Str. 9, LT-44307 Kaunas, Lithuania; agne.giedraitiene@lsmu.lt; 5 Department of Anatomy and Physiology, Veterinary Academy, Lithuanian University of Health Sciences, LT-44307 Kaunas, Lithuania; lina.merkeviciene@lsmu.lt

**Keywords:** walnut septa, by-product, antibacterial activity, amino acids, phenolic compounds, HPLC analysis, GC-MS analysis

## Abstract

Walnut septum (WS), a major by-product of walnut processing, represents a promising source of bioactive compounds with potential antioxidant and antimicrobial properties. This study aimed to characterise the phytochemical composition of WS extracts from different habitat origins and evaluate their antimicrobial activity. Total amino acids were profiled by gas chromatography–mass spectrometry, while phenolic compounds were analysed using high-performance liquid chromatography. Both methods were evaluated according to ICH Q2 (R2) guidelines for analytical procedure validation. The results showed a complex composition of amino acids and polyphenols, including ellagic acid and quercitrin. However, it was clear that habitat variations in WS samples had a significant impact on the quantities and composition of phenolic compounds and total amino acids in WS extracts. Antimicrobial activity was assessed against Gram-positive and Gram-negative bacterial strains. Variations in antimicrobial efficacy were associated with differences in phenolic composition and content due to habitat differences in WS sample origins. Collectively, this study highlights the WS as a valuable agro-industrial by-product with potential applications as a natural source of antimicrobial compounds in food and pharmaceutical systems.

## 1. Introduction

Natural by-products from the food and agriculture sectors are gaining recognition as cost-effective ways to reduce waste and support a circular economy [1,2,3,4]. They serve as sources of bioactive compounds with antioxidant and antimicrobial properties, encouraging a transition from synthetic to natural solutions and additives [5,6,7].

Walnut septum (WS), the delicate membrane that segregates the kernel, is a major by-product characterised by a structurally intricate matrix rich in secondary metabolites, which may possess potential functional and pharmacological significance [8,9,10,11]. Structurally, WS represents a lignocellulosic matrix derived from metabolically active tissues and enriched in secondary metabolites with potential functional and pharmacological relevance [12]. Due to its structural role, WS tends to accumulate ellagitannins and other hydrolysable tannins, compounds involved in plant defence mechanisms through antioxidant and antimicrobial activities [13]. Extensive research on WS [8,9,10], including our own recent study [14], has shown that WS extracts contain a complex composition of polyphenols. This corresponds to the notable antioxidant activity of WS extracts. However, comprehensive evaluations of habitat variations in phytochemical composition and their associated functional effects remain limited. Phenolic metabolite biosynthesis and accumulation in plants are strongly influenced by both abiotic and biotic stress factors, including temperature fluctuations, ultraviolet radiation, soil mineral composition, and water availability [15,16,17]. Consequently, habitat origin may significantly influence both qualitative composition and relative abundance of secondary metabolites in WS, thereby affecting biological efficiency [14]. In addition to polyphenolic constituents, amino acids may contribute to the overall biochemical matrix of plant-derived by-products [18,19,20]. However, information regarding the free amino acid fraction of walnut septa remains scarce.

The emerging issue of antimicrobial resistance substantially limits treatment options for numerous diseases. Restrictions on synthetic preservatives have heightened interest in plant-derived compounds exhibiting broad-spectrum biological effects. In particular, polyphenol-rich plant matrices are attracting attention for their diverse antioxidant and antimicrobial properties [21,22,23]. Phenolic compounds, particularly ellagitannins and flavonoids, are widely known for their antimicrobial effects [12,24,25,26] through multiple different pathways. For example, these compounds are examined for their impact on the integrity of the cytoplasmic membrane [27], chelation of essential metal ions, like Fe (II) [28], or inhibition of microbial enzymes like Sortase A in *Staphylococcus aureus* strains [29,30]. Moreover, structural differences in bacterial cell envelopes strongly influence susceptibility to polyphenolic compounds. Gram-negative bacteria possess an additional outer membrane enriched with lipopolysaccharide [31], which functions as a selective permeability barrier. This could limit the intracellular access of many polyphenols, particularly larger and/or more hydrophobic molecules [32]. In contrast, Gram-positive bacteria lack this outer membrane, resulting in a more directly exposed thick peptidoglycan layer to antimicrobial agents. Consequently, a comparative evaluation of phenolic compound-enriched walnut septa extracts against representative Gram-positive and Gram-negative strains is essential for determining the antimicrobial spectrum, selectivity, and potential structure activity relationships.

This research aimed to deepen the phytochemical analysis of WS extracts and assess their antimicrobial properties, with particular focus on samples from various regions. By integrating chromatographic and mass-spectrometric profiling with biological testing, the study explored how chemical composition relates to antimicrobial effectiveness, thus promoting WS as a valuable source of bioactive compounds for use in the food and pharmaceutical sectors.

## 2. Results and Discussion

### 2.1. Amino Acids Qualitative and Quantitative Profiling by GC-MS

During the initial GC-MS analysis of free amino acids [33] in the ethanolic extract, only four amino acids, L-leucine, L-isoleucine, L-proline, and L-valine, were detected. However, the concentrations of all amino acids were below the LOQs of the analytical procedure, preventing reliable quantitative evaluation. This result could indicate that free amino acids are present only in negligible amounts in the studied samples and that analysis of free amino acids alone is insufficient for comprehensive amino acid profiling.

**Figure 1:**
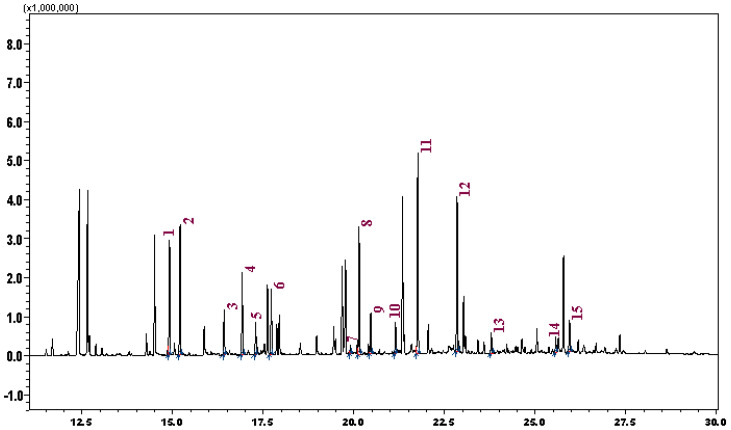
The total ion chromatogram of identified amino acids after acidic hydrolysis.

To enable the detection and quantification of total amino acids, acid hydrolysis was subsequently applied. Extract samples were treated with a 6 N HCl solution and hydrolysed in a glycerol bath at 110 °C for 5 h [34]. During acidic hydrolysis, proteins and peptide-bound amino acids were cleaved at peptide bonds, resulting in the release of individual amino acids that are otherwise present in peptides or proteins. This approach was selected to increase both the number of detectable amino acids and their concentrations, thereby allowing accurate qualitative and quantitative total amino acid profiling by GC-MS. After hydrolysis, samples were analysed by GC-MS under the same sample preparation and analysis conditions as for the non-hydrolysed samples. A representative total ion chromatogram (TIC) of hydrolysed samples is presented in Figure 1.

**Figure 2:**
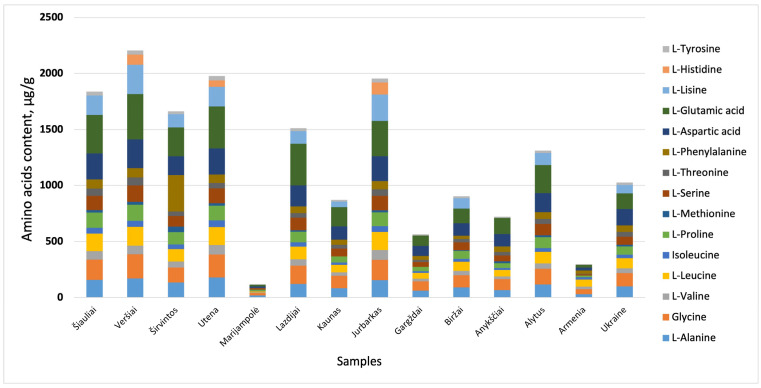
Region variations in amino acid content in walnut septa samples.

**Figure 3:**
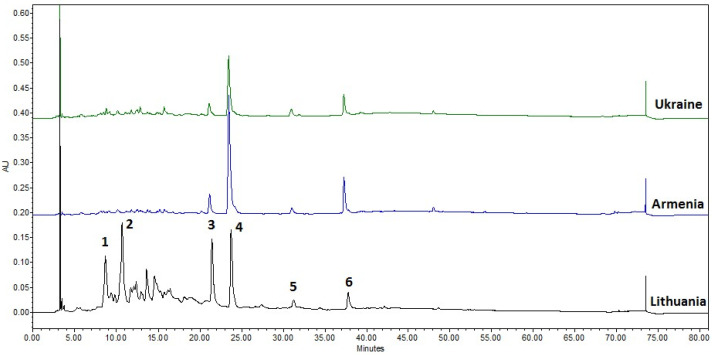
Representative chromatographic profiles of walnut septa extract samples from Ukraine, Armenia, and Lithuania.

**Figure 4:**
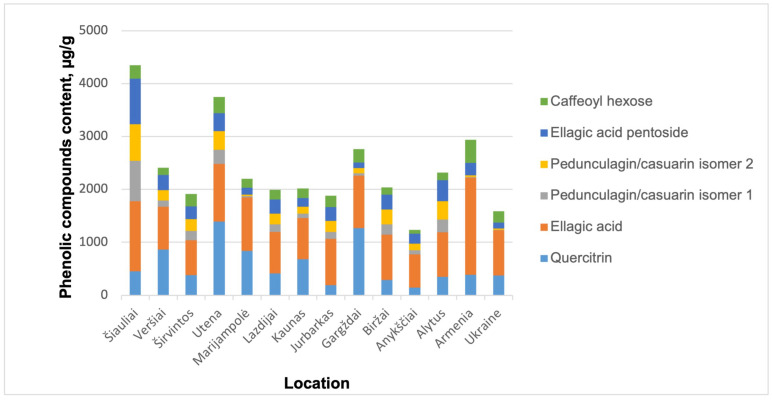
Region variations in phenolic compounds content in walnut septa samples.

**Figure 5:**
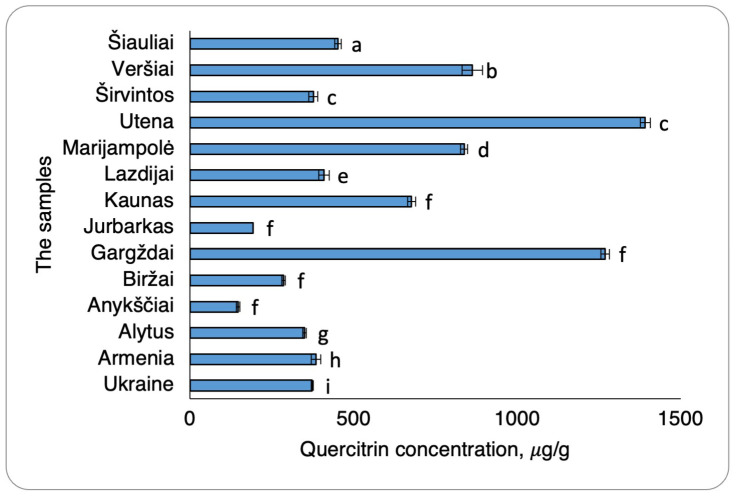
Quercitrin concentrations in Ukraine, Armenia, Lithuania walnut septa extract samples. The data are presented as the mean ± S.D. (*n* = 2). Samples were grouped according to statistically significant differences (*p* < 0.05). Samples sharing the same letter belong to the same statistical group and do not differ significantly, whereas samples with different letters represent significantly different groups.

**Figure 6:**
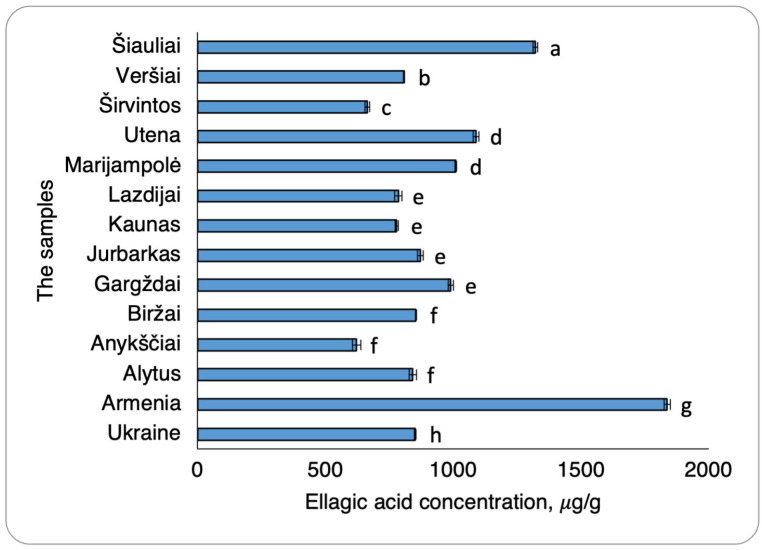
Ellagic acid concentrations in walnut septa extract samples from Ukraine, Armenia, and Lithuania. The data are presented as the mean ± S.D. (*n* = 2). Samples were grouped according to statistically significant differences (*p* < 0.05). Samples sharing the same letter belong to the same statistical group and do not differ significantly, whereas samples with different letters represent significantly different groups.

**Figure 7:**
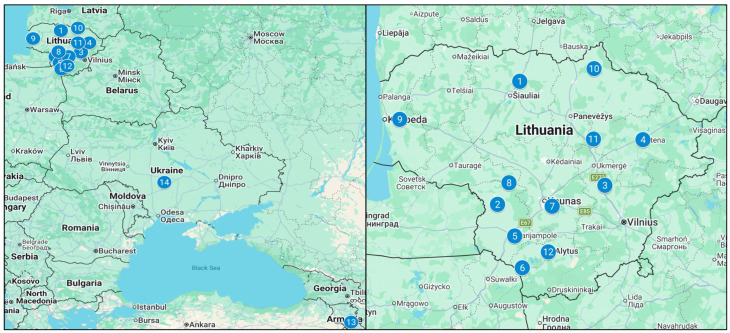
Walnut septa sample origin map, where 1—Šiauliai; 2—Šakiai district; 3—Širvintos; 4—Utena; 5—Marijampolė; 6—Lazdijai; 7—Kaunas; 8—Veliuona; 9—Gargždai; 10—Biržai; 11—Anykščiai district; 12—Alytus; 13—Armenia; 14—Ukraine.

In total, 15 amino acids were identified based on their peak’s retention times (TIC RT) and characteristic mass spectral fragmentation patterns for each detected peak. The detected amino acids and their corresponding RTs were as follows: L-alanine (14.91 min, Figure 1-1), glycine (15.21 min, Figure 1-2), L-valine (16.42 min, Figure 1-3), L-leucine (16.92 min, Figure 1-4), L-isoleucine (17.29 min, Figure 1-5), L-proline (17.72 min, Figure 1-6), L-methionine (19.90 min, Figure 1-7), L-serine (20.14 min, Figure 1-8), L-threonine (20.47 min, Figure 1-9), L-phenylalanine (21.15 min, Figure 1-10), L-aspartic acid (21.76 min, Figure 1-11), L-glutamic acid (22.85 min, Figure 1-12), L-lysine (23.79 min, Figure 1-13), L-histidine (25.57 min, Figure 1-14), and L-tyrosine (25.95 min, Figure 1-15) (Figure 1).

### 2.2. Habitat Variations in Juglans regia L. Septa Samples on the Total Amino Acids Content

Following hydrolysis of the walnut septa extracts with 6 N HCl, the samples were subsequently analysed using gas chromatography–mass spectrometry (GC-MS). Two separate injections for each sample were performed, and the results from both injections were averaged. The highest amino acid content was observed in samples collected from Veršiai, with a total amino acid concentration of 2205.07 µg/g (Figure 2).

Furthermore, it was observed that walnut septa samples collected in various regions of Lithuania contained more amino acid content than samples from Armenia (total 296.59 µg/g) (Figure 2). However, the Marijampolė (Lithuania) sample was the exception, which resulted in the lowest amounts of amino acids in all tested samples. The determined total concentration of amino acids is only 117.16 µg/g. It was noticed that most of the walnut septa samples collected in mostly northern and central Lithuania (Šiauliai, Veršiai, Širvintos, Utena, Lazdijai, and Jurbarkas) exhibited significantly higher amino acid content than their counterparts from Ukraine (1024.78 µg/g) or Armenia (Figure 2). This may be a result of differences in growing conditions or genotype [35]. A limitation of this study is the relatively small number of samples per geographical group, particularly for Armenian and Ukrainian samples. This constraint may affect the robustness of multivariate analysis; therefore, a univariate statistical approach was applied to ensure a reliable interpretation of the results. Nevertheless, multivariate methods, such as principal component analysis (PCA), could serve as valuable tools for exploring relationships between geographical origin and compositional data. Future studies should include larger datasets and a greater number of samples per group to enable more robust multivariate analysis and to better capture underlying biological and geographical variability.

### 2.3. Phenolic Compounds Composition of the Juglans regia L. Septa Extract Samples and Method Feasibility for Quantitative/Qualitative Analysis of Phenolic Compounds

An HPLC-PDA analysis of *Juglans regia* septa extract samples was performed to determine the chemical composition of the walnut septa extract samples. The following 6 compounds were identified in the walnut septa extract sample (Veršiai, Lithuania) (Figure 3): pedunculagin/casuarin isomer 1 (peak no. 1, RT 7.94 min), pedunculagin/casuarin isomer 2 (peak no. 2, RT 9.94 min), ellagic acid pentoside (peak no. 3, RT 20.71 min), ellagic acid (peak no. 4, RT 23.00 min), quercitrin (peak no. 5, RT 30.50 min), and caffeoyl hexose-deoxyhexoside (peak no. 6, RT 37.03 min).

It is known that habitat origin has a significant impact on the phytochemical composition and antioxidant capacity of *Juglans regia* L. Septa [35]. Furthermore, the walnut septa extract obtained from Lithuania was compared with corresponding samples from Ukraine and Armenia. The aim of this analysis was to evaluate the habitat distribution of phenolic compounds across Eastern Europe (Lithuania and Ukraine) and Western Asia (Armenia). The results indicated that walnut septa extracts from Ukraine and Armenia contained only trace amounts of pedunculagin/casuarin isomers 1 and 2. In the UV–Vis chromatograms, low-intensity peaks were detected within the expected retention time range for pedunculagin/casuarin isomers 1 and 2 and were assigned accordingly based on retention times and UV–Vis spectra obtained using PDA detection (Figure 3), peaks 1 and 2 in the Lithuania chromatogram, respectively). In contrast, the UV–Vis chromatogram of the Lithuanian sample exhibited pronounced, well-defined peaks corresponding to both pedunculagin/casuarin isomers 1 and 2. Moreover, although ellagic acid pentoside, quercitrin, ellagic acid, and caffeoyl hexose-deoxyhexoside were detected in all samples, their concentrations in the Armenian and Ukrainian WS extracts were significantly lower than those measured in the Lithuania sample.

### 2.4. Habitat Variations in Phenolic Compounds Profile in the Juglans regia L. Septa Extract Samples

For each sample, two independent injections were performed, and the results were averaged. The analysis revealed that the highest total phenolic compound concentrations were observed in samples from two Lithuanian regions: Šiauliai (4350.58 µg/g) and Utena (3750.26 µg/g), both significantly higher than those measured in the Armenian and Ukrainian samples (Figure 4).

The Gargždai (Lithuania) and Armenian walnut septa samples contained comparable total phenolic compound concentrations, measured at 2765.23 µg/g and 2936.24 µg/g, respectively. However, despite the slightly higher total phenolic content observed in the Armenian sample, this value was largely attributable to a disproportionately high concentration of ellagic acid, as illustrated in Figure 4. Apart from ellagic acid and quercitrin, the concentrations of the remaining identified phenolic compounds were comparatively low. A similar distribution pattern was also observed in the Ukrainian walnut septa extract sample. These elevated levels of ellagic acid observed in Armenian-grown WS may indicate that WS from this region could be a superior natural source of ellagic acid. However, further extensive studies are required to confirm this observation.

Overall, the present results indicate that Lithuanian-grown walnut septa, particularly from northern regions, may serve as a promising source of a broad spectrum of bioactive phenolic compounds. At the same time, the ellagic acid levels observed in Armenian-grown samples suggest further targeted investigation to better understand region-specific differences in metabolite accumulation in Armenia, as well as the environmental and genetic factors that may contribute to these variations.

Comparison with the available literature is constrained by the limited number of studies specifically investigating walnut septum, as well as by considerable methodological variability among published reports. Most existing studies have focused on the determination of total phenolic content using the Folin–Ciocalteu assay, with results expressed as gallic acid equivalents (GAE), whereas comprehensive chromatographic profiling of individual phenolic compounds remains scarce. For instance, Liu et al. [36] reported a total polyphenol content of 122.78 ± 2.55 mg GAE/g dry weight in walnut septum and identified 75 phenolic compounds, including flavonoids, tannins, and phenolic acids, using UPLC–Orbitrap MS, thereby demonstrating the chemical complexity of this matrix. Similarly, Delibaş and Kıray [37] reported a total phenolic content of 119.42 ± 2.39 mg GAE/g dry weight for ethanolic extracts of walnut kernel septum, further supporting its richness in phenolic constituents. Nevertheless, direct comparison across studies remains challenging due to differences in extraction solvents, extraction protocols, plant origin, and analytical methodologies. Such methodological heterogeneity should be carefully considered when interpreting discrepancies between the present findings and previously reported data. Despite these limitations, the available evidence consistently indicates that walnut septum represents a phenolic-rich plant material with significant phytochemical potential.

### 2.5. HPLC-PDA Method Feasibility for Qualitative/Quantitative Evaluation of Ellagic Acid and Quercitrin

To demonstrate the suitability of the HPLC-PDA method for the accurate and reliable quantification of ellagic acid and quercitrin in walnut septa extracts, the method was examined in accordance with the International Council for Harmonisation (ICH) Q2 (R2) guideline on validation of analytical procedures. The study was performed by assessing the following analytical performance characteristics for a quantitative test: specificity, linearity, precision (repeatability and intermediate precision), limit of detection (LOD), and limit of quantification (LOQ). Reference standards of ellagic acid and quercitrin were used for the evaluation of all validation parameters.

First and foremost, no interfering peaks in UV–Vis chromatograms were observed at the respective retention times of ellagic acid and quercitrin. Blank samples, containing mobile phases and sample matrix, did not contain any peaks in the retention time range of targeted quercitrin and ellagic acid. This showed that there is no measurement interference from the sample matrix or mobile phases. This confirmed method specificity. A linear response was achieved in a range of 0.408–23.712 µg/mL with a coefficient of determination (R^2^) ≥ 0.990 for ellagic acid, while for quercitrin it was 7.008–205.616 µg/mL with a coefficient of determination (R^2^) ≥ 0.999.

Method precision was assessed in terms of repeatability and intermediate precision. Repeatability was evaluated by analysing six replicate injections of the same concentration on the same day, using the same equipment and performed by a single operator. The relative standard deviation (RSD%) for retention time (RT) and peak area were calculated. For ellagic acid, RSD% values of 0.10% for RT and 0.25% for peak area were obtained, while for quercitrin the corresponding RSD% values were 0.10% for RT and 0.30% for peak area. Intermediate precision was evaluated by performing the analysis on different days using the same equipment and the same operator. The calculated RSD% values for ellagic acid were 0.20% for RT and 0.29% for peak area, whereas for quercitrin the RSD% values were 0.21% for RT and 0.32% for peak area. The precision studies demonstrated that the method exhibits satisfactory repeatability and intermediate precision, confirming its suitability for the quantitative determination of ellagic acid and quercitrin in walnut septa extract samples.

The limits of detection (LOD) for ellagic acid and quercitrin were determined to be 0.051 µg/mL and 0.109 µg/mL, respectively, while the corresponding limits of quantification (LOQ) were 0.211 µg/mL and 0.295 µg/mL. These results demonstrate that the analytical procedure is sufficiently sensitive and fit for its intended purpose, enabling the reliable quantitative determination of ellagic acid and quercitrin in walnut septa extract samples.

### 2.6. Habitat Comparison Study of Ellagic Acid and Quercitrin Content in the Juglans regia L. Septa Extract Samples

Since ellagic acid and quercitrin are among the most important and extensively studied phenolic compounds due to their abundance in plant-derived foods and their broad range of biological activities [38,39], it was decided to further compare habitat distribution of these compounds in walnut septa extract samples.

The determination of quercitrin concentrations across different regions demonstrated statistically significant habitat differences (Figure 5). Overall, walnut septa extracts obtained from Lithuanian regions contained higher quercitrin levels compared to those derived from Armenian and Ukrainian samples. The highest quercitrin concentrations were observed in extracts from Utena and Gargždai (northern Lithuanian regions), measuring 1391.57 ± 15.03 µg/g and 1269.43 ± 12.90 µg/g, respectively. In contrast, quercitrin concentrations in Armenian and Ukrainian walnut septa extracts were significantly lower. Notably, the lowest quercitrin concentration among all analysed samples was detected in the extract collected from Anykščiai (Lithuania), with a value of 146.58 ± 5.32 µg/g, which was the lowest in all Lithuanian samples.

The quantification of ellagic acid in walnut septa extracts obtained from different habitat regions revealed statistically significant habitat variability. The extract derived from Armenian walnut septa contained the highest ellagic acid concentration, measured at 1836.35 ± 12.30 µg/g (Figure 6). This showed that this concentration was significantly higher than those detected in the Lithuanian and Ukrainian samples. Specifically, the Ukrainian walnut septa extract exhibited an ellagic acid concentration of 851.77 ± 2.73 µg/g, representing an approximately 1.0 g/mL lower concentration compared to the Armenian sample. Lithuanian walnut septa extracts also demonstrated comparatively lower ellagic acid levels. Among these samples, the Šiauliai extract contained the highest concentration (1322.17 ± 9.22 µg/g). In contrast, extracts from Gargždai and Utena, despite exhibiting the highest quercitrin concentrations, showed only moderate ellagic acid contents, measured at 990.48 ± 9.35 µg/g and 1089.52 ± 11.20 µg/g, respectively (Figure 6).

### 2.7. The Antimicrobial Activity of WS Extract

The study was conducted using the agar dilution method, which demonstrated that the ethanolic WS extract obtained from Šiauliai (Lithuania) exhibited strong antimicrobial inhibitory activity against both *S. aureus* ATCC 25923 and *E. coli* ATCC 25922 when applied at concentrations of 2.5 mL and 5 mL per 50 mL of growth medium (Table 1). Extracts prepared from walnuts collected from two distinct habitat locations (Armenia and Ukraine) showed no inhibitory effect against *Staphylococcus aureus* and *Escherichia coli*. The antimicrobial activity of WS extracts demonstrated a concentration-dependent inhibitory effect, where higher extract volumes resulted in increased suppression of bacterial growth. This trend supports the biological relevance of the observed effects despite the qualitative scoring system.

Since the only ethanolic WS extract obtained from Šiauliai (Lithuania) exhibited inhibitory activity against the two test bacterial cultures, further experiments to determine the effect of this extract were conducted using additional cultures, *Bacillus subtilis* ATCC 6633, *Bacillus cereus* ATCC 10876, *Enterococcus faecalis* ATCC 29212, and *Pseudomonas aeruginosa* ATCC 9027.

Table 2 illustrates differences in the antimicrobial activity of the ethanolic WS extract from Šiauliai (Lithuania) against the tested microorganisms. It was determined that this WS extract does not affect *Bacillus subtilis* ATCC 6633, *Bacillus cereus* ATCC 10876, *Enterococcus faecalis* ATCC 29212, and *Pseudomonas aeruginosa* ATCC 9027 growth when applied at concentrations of 2.5 mL and 5 mL per 50 mL of growth medium.

Most evidence for antimicrobial activity of the WS was derived from in vitro studies, and its effectiveness depends strongly on the extraction method (aqueous or alcoholic), extract concentration, and the type of microorganism tested.

This study indicates that the antibacterial activity of an ethanolic extract of WS was influenced by both the concentration of extract applied to the growth medium and the geographic origin of the walnuts. Using the agar dilution method, the results demonstrated that an extract concentration of 2.5 mL per 50 mL of medium was sufficient to exhibit antimicrobial activity against the Gram-negative bacterium *Escherichia coli* and the Gram-positive bacterium *Staphylococcus aureus*. Our previous study found significant habitat differences in phenolic composition of walnut septa, with samples from Šiauliai (Lithuania) showing higher phenolic concentrations and antioxidant activity, indicating that geographic origin influences bioactive compound profiles [14]. The present study corroborates earlier findings, demonstrating that the WS extract obtained from Šiauliai, Lithuania, exhibited greater antimicrobial activity compared to extracts derived from Ukraine. Notably, WS extracts from Armenia, despite containing elevated levels of ellagic acid, showed lower antimicrobial efficacy than the extracts prepared from walnuts collected in Šiauliai. These results suggest that antimicrobial activity may not be attributable to ellagic acid; rather, it is likely influenced by synergistic interactions among multiple phenolic constituents [40]. Furthermore, Šiauliai WS extract samples contained substantial levels of pedunculagin/casuarin isomers and ellagic acid pentoside, compared with Armenian samples. Ellagitannins are known for their disruption of bacterial walls and interference with enzymatic systems of bacteria [41]. Their presence in higher concentrations may significantly contribute to the enhanced antimicrobial effect. The limitation of this study is the qualitative nature of antimicrobial assessment; however, this reflects its design as a preliminary screening study rather than a quantitative analysis. Future studies should include MIC determination to provide a more precise evaluation of antimicrobial potency.

According to other studies, the methanolic extract of WS had strong antibacterial activity, showing the highest efficacy against *S. aureus* [42]. Genovese et al. [43] showed that alcoholic extracts of WS had strong antibacterial activity against Gram-positive bacteria (e.g., *Staphylococcus aureus*, *S. epidermidis*, *Enterococcus faecalis*, *E. faecium*) and were less inhibitory against Gram-negative strains (e.g., *Escherichia coli*, *Klebsiella pneumoniae*, *Pseudomonas aeruginosa*, *Proteus mirabilis*) at comparable extract concentrations, as indicated by lower minimum inhibitory concentration (MIC) values for Gram-positive bacteria. To conclude, additional mechanistic investigations are required to clarify the exact pathways underlying the observed antimicrobial effects.

## 3. Materials and Methods

### 3.1. Plant Materials and Reagents

Walnut (*Juglans regia* L.) fruits were collected in autumn 2024 (late September to early October) from multiple habitats across Lithuania, including Šiauliai (56.07331, 23.46924), Šakiai district (54.86087, 23.07875), Širvintos (55.05227, 24.94420), Utena (55.50453, 25.59484), Marijampolė (54.54829, 23.37683), Lazdijai (54.22736, 23.51041), Kaunas (54.84244, 24.02246), Veliuona (55.07759, 23.27818), Gargždai (55.70520, 21.39497), Biržai (56.19480, 24.75569), Anykščiai district (55.50758, 24.74563), and Alytus (54.38703, 23.96509) (Figure 7). In each habitat, fruits were collected from two mature, ungrafted walnut trees of unknown cultivar growing in homestead environments. Approximately 2 kg of fruits were collected per site. Fruits were harvested at full maturity after natural abscission, ensuring that only fully ripe material was used. After collection, the fruits were dried at room temperature (approximately 20–22 °C) for 2–3 weeks until constant moisture content was achieved. The shells were then opened, and the septa were manually separated. The obtained septa were milled using an Ultra Centrifugal Mill ZM 200 (Retsch, Haan, Germany) equipped with a 0.25 mm sieve. The processed material was stored in glass containers in a dark, dry place at room temperature until further analysis.

Walnut samples from Ukraine and Armenia were obtained from single growing areas in each country and were processed following the same protocol as described for Lithuanian samples.

To obtain a more comprehensive understanding of the regional distribution of phenolic compounds, walnut septa extract samples collected from various Lithuanian regions (Šiauliai, Veršiai, Širvintos, Utena, Marijampolė, Lazdijai, Kaunas, Jurbarkas, Gargždai, Biržai, Anykščiai, and Alytus) were compared with samples from Armenia and Ukraine by GC-MS and HPLC-PDA methods.

Ethanol (96%) Vilniaus degtine (Vilnius, Lithuania). Purified water and deionised water were prepared with GFL2004 (GFL, Burgwedel, Germany) and Millipore SimPak 1 (Merck, Darmstadt, Germany).

Acetonitrile, methanol, and acetone were obtained from Fisher Scientific (Waltham, MA, USA). MTBSTFA was obtained from Sigma-Aldrich (St. Louis, MO, USA). Hydrochloric acid (HCl) and glycerol were obtained from Merck (Darmstadt, Germany). Trifluoroacetic acid, diphenyl and dimethylpolysiloxane were obtained from Sigma-Aldrich (St. Louis, MO, USA). Pedunculagin/casuarinin isomer I (bis-HHDP-glucose), pedunculagin/casuarinin isomer II (bis-HHDP-glucose), ellagic acid pentoside, and caffeoyl hexose-deoxyhexoside were obtained from Sigma-Aldrich (St. Louis, MO, USA) and Amino Acids Standard Mixture (Sigma-Aldrich, Schnelldorf, Germany).

### 3.2. Sample Preparation

#### 3.2.1. Sample Preparation Without Hydrolysis for GC-MS

The extract for analysis was done using 0.1 g of plant material with 10 mL of 70% methanol in an ultrasonic bath (frequency of 38 kHz) (Grant Instruments™ XUB12 Digital, Cambridge, UK) at room temperature (20 ± 2 °C) for 15 min. The extract was centrifuged at 5000 rpm (Centurion Scientific, model C206, Stoughton, UK) for 10 min at 25 °C. An aliquot of 500 µL of the supernatant was evaporated to dryness under a nitrogen stream. The residue was reconstituted in 100 µL of acetonitrile and 100 µL of MTBSTFA and derivatised at 100 °C for 2.5 h [33,44]. A volume of 1 µL was injected into the GC-MS system.

#### 3.2.2. Acidic Hydrolysis and Sample Preparation for GC-MS

For hydrolysed samples, the extract was prepared using 1 g of plant material that was treated with 10 mL of 6 N HCl and hydrolysed in a glycerol bath at 110 °C for 5 h. After cooling, the hydrolysate was centrifuged at 5000 rpm for 15 min at 25 °C. A 100 µL aliquot of the supernatant was evaporated under nitrogen, reconstituted in 100 µL of acetonitrile and 100 µL of MTBSTFA, and derivatised at 100 °C for 2.5 h. A volume of 1 µL was injected into the GC-MS system.

### 3.3. GC-MS Analysis

#### 3.3.1. GC-MS Conditions

GC-MS analysis was performed using a Shimadzu GC/MS-QP2010 Ultra system equipped with an electron ionisation (EI) source and a split/splitless injector. Chromatographic separation was achieved on an Rxi-5 ms capillary column (30 m × 0.25 mm, 0.25 µm film thickness; 5% diphenyl/95% dimethylpolysiloxane). Helium (99.999%) was used as the carrier gas at a constant flow rate of 1.49 mL/min.

The oven temperature program was set to 75 °C (5 min), increased to 290 °C at 10 °C/min, then to 320 °C at 20 °C/min and held for 10 min. The injector temperature was maintained at 250 °C, and the injection volume was 1 µL. The MS interface and ion source temperatures were set at 280 °C and 200 °C, respectively. The mass spectrometer was operated in positive EI mode at 70 eV. Full-scan acquisition (*m*/*z* 35–500) was used for compound identification, while quantitative analysis was performed in selected ion monitoring (SIM) mode. Data acquisition and processing were carried out using LabSolutions GC/MS software (Version 5.71). Identification of volatile compounds was carried out using mass spectra library search (NIST 14) and compared with the acquired MS data of the standards and mass spectral data from the literature [45].

#### 3.3.2. GC-MS Method Feasibility

To demonstrate methods’ feasibility for accurately and reliably determining walnut septa extract samples’ amino acid qualitative and quantitative profiles by GC-MS, it was decided to evaluate the method according to ICH Q2 (R2) guidelines for analytical procedure validation. Typical Analytical Procedure Performance Characteristics for quantitative methods were selected: specificity, linearity, precision (repeatability and intermediate precision), limit of detection (LOD), and limit of quantification (LOQ).

No interfering peaks in TIC were observed at the respective retention times of amino acid peaks. In each mass spectrum of amino acid TIC peaks, no overlapping unidentified ions with high intensity were found, showing only characteristic fragmentation patterns of each amino acid TBDMS derivative. Furthermore, the blank sample did not contain any peaks in the retention time range of targeted amino acids, which showed that there is no measurement interference from the sample matrix. This confirmed that there were no overlapping peaks in TIC of the samples, confirming method specificity. Linear response was achieved in a range of 25.0–200.0 µg/mL with coefficients of determination (R^2^) ≥ 0.980 for all analytes in question.

Method precision was evaluated by performing repeatability and intermediate precision experiments. During repeatability evaluation, 6 injections at the same concentration were analysed on the same day and equipment by one operator. A relative standard deviation (RSD%) between injections for peak areas and RT was calculated, yielding values below 2.5% and 1%, respectively, for all analytes. Intermediate precision was evaluated by performing the analysis on different days while using the same equipment by the same operator. The relative standard deviation (RSD%) between the injections for peak areas and peak RT was calculated, yielding values below 1% (for RT) and below 2.5% (for peak areas) across the experiments. Precision experiments demonstrated adequate repeatability and intermediate precision of the method for quantitative amino acid analysis of walnut septa extract samples. The determined LOD value for all analytes in the samples was below 3.0 µg/mL, while the determined LOQ value was below 10.0 µg/mL. These experiments confirmed that the analytical procedure is fit for the intended use to qualitatively and quantitatively assess the amino acid profiles of walnut septa extract samples.

### 3.4. HPLC-PDA Analysis

HPLC-PDA analysis was carried out using a Waters e2695 separation module coupled with a Waters 2998 photodiode array detector. Chromatographic separation was performed on an ACE C18 column (250 mm × 4.6 mm, 5.0 µm). The mobile phase consisted of solvent A (0.1% trifluoroacetic acid in water) and solvent B (acetonitrile), delivered at a constant flow rate of 1.0 mL/min.

Gradient elution was applied as follows: 95% A/5% B (0 min), 85% A/15% B (8 min), 80% A/20% B (30 min), 50% A/50% B (58–65 min), 5% A/95% B (66–70 min), followed by re-equilibration to initial conditions at 71 min. The column temperature was maintained at 25 °C, the injection volume was 10 µL, and detection was performed in the wavelength range of 200–400 nm.

Phenolic compounds were tentatively identified based on retention times and UV–Vis spectra obtained from PDA detection and by comparison with retention times of reference standards analysed under the same conditions.

### 3.5. Antimicrobial Activity Assays

#### 3.5.1. Microorganisms and Culture Conditions

The antimicrobial activity of ethanolic WS extracts was evaluated against Gram-positive bacteria *Staphylococcus aureus* ATCC 25923, *Bacillus subtilis* ATCC 6633, *Bacillus cereus* ATCC 10876, and *Enterococcus faecalis* ATCC 29212 and Gram-negative bacteria *Escherichia coli* ATCC 25922 and *Pseudomonas aeruginosa* ATCC 9027. The selected bacteria were chosen based on their clinical relevance and Gram classification, which allows evaluation of the antimicrobial potential of the WS extracts against both Gram-positive and Gram-negative organisms.

The microorganisms were obtained from the Institute of Microbiology and Virology, the Lithuanian University of Health Sciences, Lithuania. The bacterial strains *S. aureus*, *B. subtilis*, *B. cereus*, *E. faecalis*, and *P. aeruginosa* were cultivated on Columbia agar supplemented with 5% sheep blood (E&O Laboratories, Bonnybridge, UK), while *E. coli* was cultivated on MacConkey agar (Liofilchem, Roseto degli Abruzzi, Italy). The bacteria were incubated aerobically at +36 °C for 24–48 h.

#### 3.5.2. Preparation of the Media and Bacterial Suspension

WS extracts were prepared from walnuts collected from three geographically distinct locations: Armenia, Ukraine, and Lithuania (Šiauliai). Different volumes of the extracts (1, 2.5, and 5 mL) were aseptically transferred into the sterile graduated cylinder and thoroughly mixed. Different volumes (1, 2.5, or 5 mL) of WS extracts were incorporated into liquid Mueller–Hinton agar before further experimentation. The solid mixed medium was used to perform the agar dilution method.

Bacterial suspensions were prepared from 24 h old cultures in physiological saline and standardised using the McFarland turbidity standard. The suspensions were adjusted to 0.5 McFarland, corresponding to approximately 1.5 × 10^8^ CFU/mL, using a McFarlandometer DEN-1B (Biosan, Riga, Latvia).

A control medium without WS extract was prepared under identical conditions and used as a negative control to confirm normal bacterial growth. In addition, a reference antimicrobial agent (e.g., a standard antibiotic commonly used in susceptibility testing) was included as a positive control to verify bacterial sensitivity and assay validity.

#### 3.5.3. Agar Dilution Method

Bacterial suspensions of *Staphylococcus aureus* ATCC 25923, *Escherichia coli* ATCC 25922, *Bacillus subtilis* ATCC 6633, *Bacillus cereus* ATCC 10876, *Enterococcus faecalis* ATCC 29212, and *Pseudomonas aeruginosa* ATCC 9027 were inoculated onto Petri dishes containing Mueller–Hinton agar supplemented with different volumes (1, 2.5, or 5 mL) of WS extracts. Plates without extract served as growth (negative) controls, while plates containing the reference antimicrobial agent served as positive controls. Standardised bacterial suspensions adjusted to 0.5 McFarland were evenly spread over the surface of the solidified agar using a sterile swab. The plates were incubated at 36 °C for 24 h, after which the antimicrobial effect was evaluated. The growth of microorganisms was assessed the following day. The inclusion of both positive and negative controls ensured the reliability and interpretability of the antimicrobial assay.

The antimicrobial activity evaluation was based on the presence or absence of visible bacterial growth on agar plates, allowing a semi-quantitative assessment of inhibitory effects across different extract concentrations.

### 3.6. Statistical Analysis

Data were analysed using SPSS version 20.0 (IBM Corporation, Armonk, NY, USA). Comparisons between three different measurements were made using the Friedman and Wilcoxon tests. Statistical differences among samples for HPLC-PDA results were evaluated using one-way ANOVA followed by Tukey’s post hoc test, and results are presented using letter-based grouping, where identical letters indicate no significant differences (*p* < 0.05).

## 4. Conclusions

WS are considered a promising agro-industrial by-product with potential applications as a natural source of antioxidant and antimicrobial compounds. Although Armenian samples contained the highest levels of ellagic acid, their reduced antimicrobial activity indicates that bioactivity may result from synergistic interactions among multiple phenolic compounds rather than from a single constituent.

A concentration of 2.5 mL of ethanolic WS extract per 50 mL of medium was sufficient to demonstrate antimicrobial effects against the Gram-negative bacterium *Escherichia coli* and the Gram-positive bacterium *Staphylococcus aureus*. Differences in antimicrobial activity were observed among samples of different geographical origin; however, these should be interpreted with caution. Lithuanian WS, particularly those from northern regions (such as Šiauliai), showed the most pronounced antimicrobial effect, whereas samples from Armenia and Ukraine exhibited weaker or no inhibitory effects under the tested conditions. Given the limited number of samples and the qualitative nature of the antimicrobial assessment, these findings indicate possible trends rather than definitive conclusions regarding geographical influence. Further research is required to elucidate the mechanisms underlying their antimicrobial effects and the region-specific variations in metabolites. Nonetheless, these byproducts may be utilised in nutraceutical products or cosmetic formulations.

## Figures and Tables

**Table 1:** Results of antimicrobial activity of extracts against *S. aureus* and *E. coli* using the agar dilution method.

Origin of Walnut Septum Extract	Concentration (mL/50 mL of Medium or %)	Growth of Bacterial Culture	
		*Staphylococcus aureus* ATCC 25923	*Escherichia coli* ATCC 25922
Armenia (1)	1 mL of extract in 50 mL of medium	+	+
Ukraine		+	+
Lithuania, Šiauliai		+	+
Armenia	2.5 mL of extract in 50 mL of medium	+	+
Ukraine		+	+
Lithuania, Šiauliai		−	−
Armenia	5 mL of extract in 50 mL of medium	+	+
Ukraine		+	+
Lithuania, Šiauliai		−	−

“+”—indicates no inhibition of bacterial growth; “−”—indicates inhibition of bacterial growth.

**Table 2:** Results of the antimicrobial activity of ethanolic WS extract from Šiauliai (Lithuania) against other microorganisms using the agar dilution method.

Origin of Walnut Septum Extract	Ethanolic Walnut Septum Extract Šiauliai (Lithuania, Concentration)	
	Concentration—2.5 mL Extract in 50 mL of Medium	Concentration—5 mL Extract in 50 mL of Medium
*Staphylococcus aureus* ATCC 25923	−	−
*Escherichia coli* ATCC 25922	−	−
*Pseudomonas aeruginosa* ATCC 9027	+	+
*Bacillus subtilis* ATCC 6633	+	+
*Bacillus cereus* ATCC 10876	+	+

“+”—indicates no inhibition of bacterial growth; “−”—indicates inhibition of bacterial growth.

## Data Availability

Data are contained within the article.

## Abbreviations

The following abbreviations are used in this manuscript:

WS	Walnut septa
HPLC	High-performance liquid chromatography
HPLC-PDA	High-performance liquid chromatography–photodiode array detector
ATTC	American Type Culture Collection
GC-MS	Gas chromatography–mass spectrometry
EI	Electron ionisation
SIM	Selective ion monitoring
MS	Mass spectrometry
MTBSTFA	*N*-(*t*-butyldimethylsilyl)-*N*-methyltrifluoroacetamide
TBDMS	*tert*-Butyldimethylsilyl group
TIC	Total ion chromatogram
UV–Vis	Ultraviolet-visible light/spectroscopy
ICH	International Council for Harmonisation
PCA	Principal component analysis
RT	Retention time
RSD%	Relative standard deviation
LOD	Limit of detection
LOQ	Limit of quantitation
PDA	Photodiode array detector
